# Is It Found?—"Naboli"

**Published:** 1880-06

**Authors:** 


*Is It Found?*—"Näbolï" is the rather fanciful name given a new local anæsthetic by Dr. H. E. Dennett, of Boston, by which, it is claimed, the pain of sensitive dentine is so modified or destroyed as to render painless what was the terror of dentistry, to patients at least. The Boston *Commonwealth* contains the advertisement of the "Dennett Näbolï Company," with certificates from nearly thirty dentists of New England, in praise of this new compound.

From the appended extract from the proceedings of the Massachusetts Dental Society, published in the Boston *Daily Evening Traveller*, it would seem that that Society is rather severe on "Näbolï;" although the "Company" publish in a subsequent issue of the same paper a letter from the president of the meetings of the Society at which these same strictures were passed in which he says:

"I *know* this remedy, if properly applied, is effectual, and I know that it cannot by any possibility do injury to the tooth or patient; aud I feel sure if dentists everywhere adopt its use, their patients will not only be grateful to them but to you also for having placed it within their reach.

As to the propriety of your patenting this discovery, there will be a difference of opinion among our professional brethren. But as I knew nothing of the *intention* or the *fact* till it was accomplished, I have only to submit and comply with such terms as your 'Näbolï' Company demand, as I cannot practice dentistry with the least degree of satisfaction without it."

Here is what the Society said about it.

"At the late session of the Massachusetts Dental Society, the subject of "Näbolï," the new so-called anæsthetic, was taken up. The conduct of certain members of the Society in adopting its use and publishing advertisements or issuing cards or circulars intimating or explicitly stating that they had superior facilities for dental operations by reason of having purchased the right to use the article, was deemed unprofessional, and articles of impeachment were presented against three of the dentists, one of Boston, another of Salem and another of New Bedford. The ground of impeachment alleged was two-fold; 1st, That to advertise in any "quackish" manner by intimating the possession of superior facilities, is contrary to the by-laws of the society; and, secondly, that the Näbolï is a preparation of no scientific merit, but is compounded of articles which have long been in use either separately or in combination, and that its sale and use are urged by the manufacturers of it as a speculation on their part. It was also alleged that the preparation often fails to produce anæsthetic effects, and that there is no good sanction for its use. These points and various side issues were debated in a very lively manner, and the following resolutions were passed:

*Resolved*, That it is the duty of every dentist to understand the nature of all the materials and medicines which they use or are to use, before placing them in the mouths of their patients, and that any medicine, the formula of which is kept secret, is not worthy of their confidence or the confidence of the public.

*Resolved,* That the analysis of "Näboli," made by able and competent chemists, show that it is no new discovery, but is composed of agents at the command of each dentist, and that no license is needed to use such old and well-known medicines.

*Resolved,* That the Massachusetts Dental Society, after a long discussion, are of the opinion that the "Dennett' Dental Näboli Company" is a stock jobbing concern, designed to impose upon the public and blackmail the profession.

Under the rules no action can be taken on the impeachment articles till next December."

How far these opinions would be modified by "Näboli" being made as free as the "rubber dam," we have no means of judging; but trust that at last a real pain obtunder for dentine is discovered.

